# Identification of DNA lesions using a third base pair for amplification and nanopore sequencing

**DOI:** 10.1038/ncomms9807

**Published:** 2015-11-06

**Authors:** Jan Riedl, Yun Ding, Aaron M. Fleming, Cynthia J. Burrows

**Affiliations:** 1 Department of Chemistry, University of Utah, 315S 1400 East, Salt Lake City, Utah 84112-0850, USA

## Abstract

Damage to the genome is implicated in the progression of cancer and stress-induced diseases. DNA lesions exist in low levels, and cannot be amplified by standard PCR because they are frequently strong blocks to polymerases. Here, we describe a method for PCR amplification of lesion-containing DNA in which the site and identity could be marked, copied and sequenced. Critical for this method is installation of either the dNaM or d5SICS nucleotides at the lesion site after processing via the base excision repair process. These marker nucleotides constitute an unnatural base pair, allowing large quantities of marked DNA to be made by PCR amplification. Sanger sequencing confirms the potential for this method to locate lesions by marking, amplifying and sequencing a lesion in the *KRAS* gene. Detection using the α-hemolysin nanopore is also developed to analyse the markers in individual DNA strands with the potential to identify multiple lesions per strand.

## Abstract (web summary)

Genomic DNA lesions exist in low levels and cannot be amplified by standard PCR. Here, Riedl *et al*. report a method to amplify damaged DNA sites by replacing them via DNA repair with unnatural base pairs, which are subsequently identified by Sanger sequencing or α-hemolysin nanopore sequencing.

## Graphical abstract

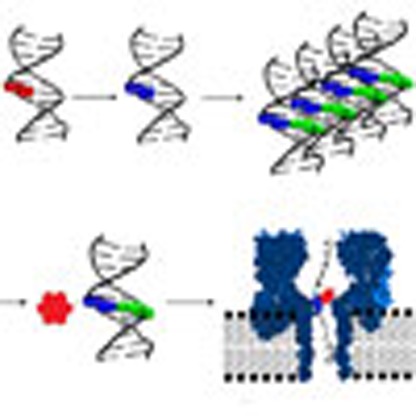

DNA undergoes damage caused by oxidation, deamination or alkylation leading to the formation of various base lesions, and depurination leading to abasic sites^1^,^2^,^3^. The location in the genome in which the modification occurs is of critical interest, because it enables understanding the origin of genetic mutations resulting from these lesions. Mutations in the genome are one hallmark of melanoma, hepatic and lung carcinomas; mutations increase with age, and are observed in a number of stress-induced disorders, such as amyotrophic lateral sclerosis^4^,^5^. A method capable of identifying the chemical identity and location in which lesions appear is crucial for determining the molecular aetiology of these diseases. Moreover, recent research has highlighted lesions in close proximity to one another to be a challenge to the repair machinery and potentially more mutagenic^6^. However, due to the low abundance of DNA modifications in the genome, it is challenging to address these questions because the damage sites display both altered base pairing and frequently are pause or stop sites for polymerases, making them unamplifiable by PCR.

A number of methods have been developed to sequence epigenetic modifications, all of which rely on conversion of the modified base to a different, but readable base, such as the bisulfite conversion of cytosine to uracil, in contrast to the chemical stability of 5-methylcytosine^7^,^8^. Variations of this method have been developed to sequence 5-hydoxymethylcytosine, 5-formylcytosine and 5-carboxycytosine using a combination of enzymatic and chemical approaches in tandem with bisulfite sequencing^7^,^8^. Direct sequencing of epigenetic modifications has been demonstrated by single-molecule real-time sequencing (SMRT) and protein nanopores^9^,^10^,^11^,^12^; though, both direct methods are challenged when working with actual tissue samples that contain modifications in low abundance.

In contrast to epigenetic modifications, DNA lesions such as those resulting from oxidative stress are diverse, and selective chemistry for them has not been developed. SMRT sequencing^13^ and ligation-mediated PCR provide limited advances in identifying base modifications^14^. An approach that retains the lesion location while providing a detectable signal for multiple lesions in proximity would enhance our understanding of lesions in the genome and how they contribute to mutagenesis.

An approach for mapping the precise location and identity of the lesion in DNA strands would be to label the damaged site with a marker nucleotide triphosphate during polymerase extension. The first generation of this approach attempted modified nucleotide insertion opposite an abasic site (AP)^15^,^16^,^17^. These approaches suffered from the inability to extend past the site at which the marker was placed and were only applicable to abasic sites (AP sites). In addition, outcompeting insertion of dATP opposite an abasic site (that is, the ‘A rule') with a modified nucleotide is challenging^18^. In a second generation for labelling lesion sites with a marker nucleotide, the Sturla laboratory demonstrated insertion and linear extension of a marker nucleotide opposite *O*^6^-benzylguanines^19^,^20^,^21^. In this report, we developed a third generation approach for labelling lesion sites with an amplifiable marker nucleotide. This method utilizes the base excision repair (BER) pathway to target lesions to yield gaps in the DNA to only insert the marker nucleotide bypassing the ‘A rule'; these markers are exponentially PCR amplifiable; and lesion identity is determined by the BER enzymes avoiding lengthy synthesis of lesion-specific markers.

Critical for the development of our method is utilization of a marker nucleotide that has a selective, complementary partner allowing high-fidelity PCR amplification of the marked DNA. For this purpose, we chose the dNaM or dMMO2 nucleotides base paired with d5SICS that form an established set of unnatural base pairs (UBPs; Fig. 1)^22^,^23^,^24^. These UBPs combined with the selectivity of the BER process allow insertion of the marker at the lesion site with excellent retention during PCR amplification, as described below.

Sequencing the UBPs is determined by a sharp stop in a Sanger sequencing chromatogram causing loss of all downstream information^22^. Therefore, to map more than one modification site per DNA strand, the dNaM nucleotide is also utilized to install an orthogonal functional group for attaching a detectable functionality^25^. The readout for this adduct is achieved using the α-hemolysin (α-HL) nanopore that analyses individual DNA molecules while they are electrophoretically driven through the small aperture pore^26^,^27^. Our concept is developed around common lesions found in the genome that include an abasic site (depurination lesion), 2′-deoxyuridine (dU, deamination lesion), as well as 8-oxo-7,8-dihydro-2′-deoxyguanosine (dOG) and spiroiminodihydantoin-2′-deoxynucleoside (dSp, oxidative lesions).

## Results

### Outline of labelling methodology

**Figure 1:**
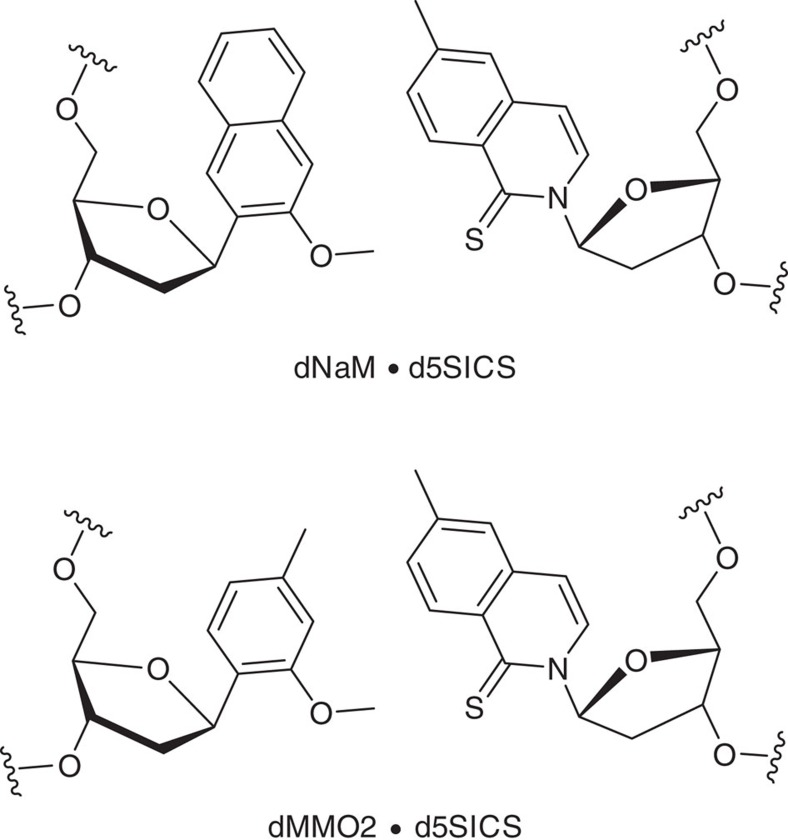
Unnatural base pairs used for labelling of DNA lesions. The dNaM·d5SICS and dMMO2·d5SICS unnatural base pairs utilized for labelling DNA lesions with a third, amplifiable marker base pair.

**Figure 2:**
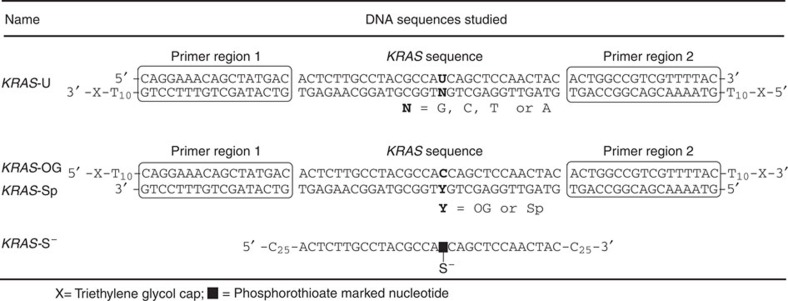
DNA sequences studied based on codon 12 of the *KRAS* gene. The *KRAS*-U duplex contains a dU in the template strand at the centre of codon 12 in the *KRAS* gene. The *KRAS*-OG and *KRAS*-Sp duplexes contain dOG or dSp, respectively, in the coding strand at the centre of codon 12. The *KRAS*-U, *KRAS*-OG and *KRAS*-Sp duplexes are flanked by primer regions that allow their PCR amplification. The *KRAS*-S^−^ strand centrally locates the *KRAS* sequence with a phosphorothioate at the centre of codon 12; this sequence also has 25-mer poly-dC tails to help facilitate entry of this strand into the α-HL nanopore.

**Figure 3:**
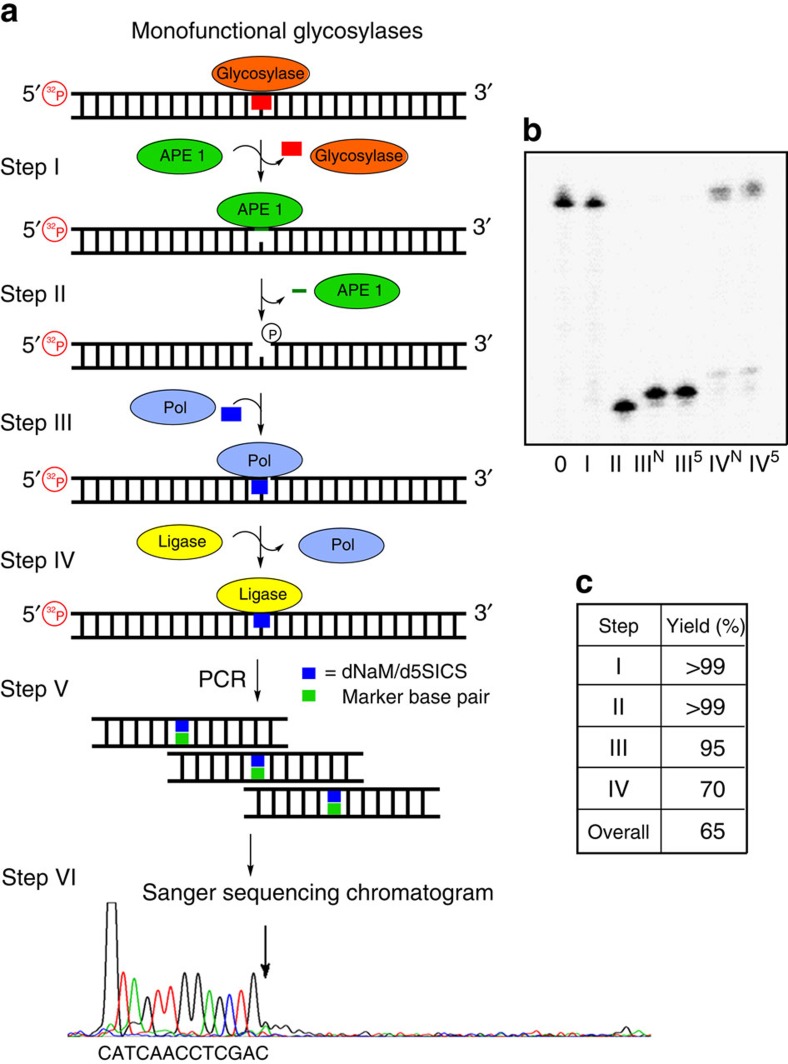
Labelling of lesions that are substrates for monofunctional BER enzymes. (**a**) The six-step scheme for labelling, amplifying and sequencing a lesion by Sanger sequencing with a template dNaM. (**b**) PAGE analysis to monitor and quantify enzyme-catalysed labelling reactions on the *KRAS*-U duplex with a dU·dG base pair (Fig. 2). Reactions were monitored by 5′ ^32^P-labelling of the lesion-containing strand, and the gels were quantified by phosphorimager autoradiography. Step 0 represents the starting strand before reaction. Steps III and IV were conducted with either dNaM or d5SICS and are marked with either a superscript N or 5, respectively. (**c**) The percentage of yield for each enzymatic step of the labelling scheme with a template dG.

**Figure 4 f4:**
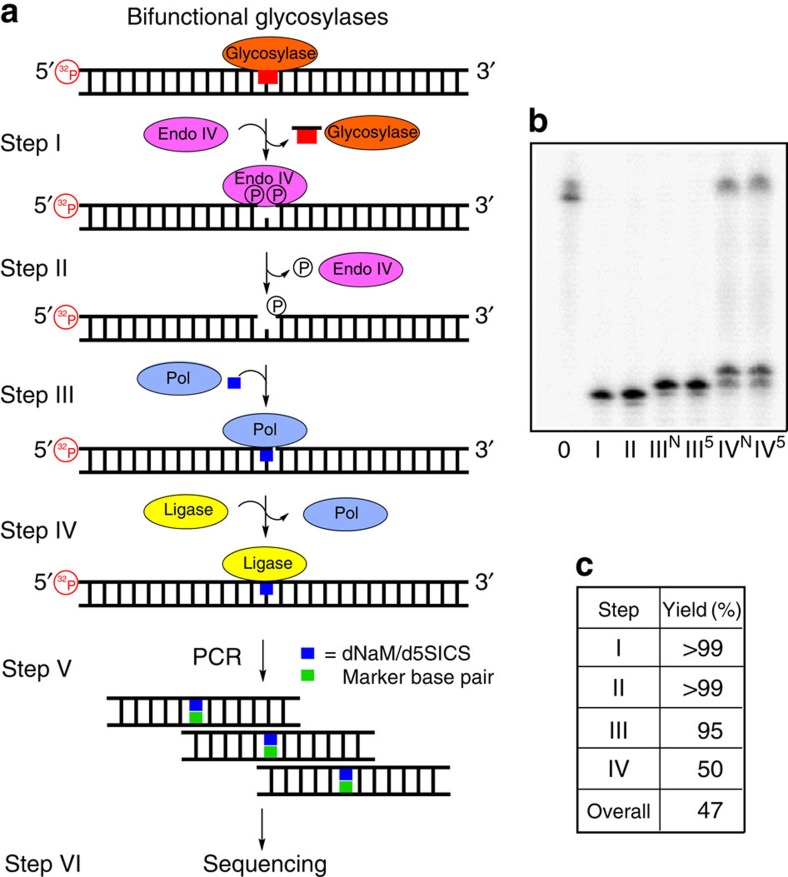
Labelling of lesions that are substrates for bifunctional BER enzymes. (**a**) The six-step scheme for labelling, amplifying and sequencing a lesion. (**b**) PAGE analysis to monitor and quantify enzyme-catalysed labelling reactions on the *KRAS*-OG duplex with Fpg (Fig. 2). Reactions were monitored by 5′ ^32^P-labelling of the lesion-containing strand, and the gels were quantified by phosphorimager autoradiography. Step 0 represents the starting strand before reaction. Steps III and IV were conducted with either dNaM or d5SICS, in which these differences are marked with either a superscript N or 5, respectively. (**c**) The percentage of yield for each enzymatic step of the labelling scheme with a template dG.

The methodology for labelling lesion sites in a DNA duplex was established in a section of the *KRAS* gene surrounding codon 12, and then applied for lesion detection in a plasmid. A G→T transversion in the coding strand of this gene in lung cancer is proposed to result from G oxidation^28^. In addition, a G→A transition found in codon 12 in this gene found in colon cancer is proposed to result from dC deamination to dU^4^. The 65-mer model system housed 30 nucleotides centred on codon 12 of the *KRAS* sequence flanked by two 17-mer PCR primer regions (Fig. 2). The non-lesion-containing strand was capped with 10 dT nucleotides terminated with triethylene glycol groups to prevent unwanted ligation reactions during one of the steps below due to the presence of a 5′-phosphate required for radioactive labelling; these tails also allowed gel purification of one strand from the other (Fig. 2). The labelling protocol is a one-pot, four-step reaction sequence that harnesses the enzymes found in the BER pathway for recognizing lesion sites and replacing them with a marker nucleotide, dNaM, d5SICS or dMMO2 (Fig. 1). The marked strand was exponentially PCR amplified with the appropriate 2-deoxynucleotide triphosphates (dNTPs) to yield UBP amplicons that were sequenced to identify the location of the lesion (Figs 3a and 4a). The complete details of each step follow.

### Site selective removal of the lesion

The selectivity of lesion labelling was defined by this step (step I; Figs 3a and 4a). As examples, four types of lesions were selected for labelling with either the dNaM or d5SICS marker nucleotides: an abasic site (AP site), a product of spontaneous depurination^29^; dU, a deamination product^30^; dOG, an oxidation product^31^,^32^; and dSp, a hyperoxidized guanine lesion^31^,^33^. These lesions have been implicated in the induction of DNA mutations found in liver and melanoma cancers^1^,^31^. All duplexes studied included site-specific lesions incorporated at known locations by solid-phase synthesis or by literature protocols (Supplementary Methods)^34^. The dU and AP site lesions were placed in the template strand of the *KRAS* sequence, while dOG and dSp were placed in the coding strand of the *KRAS* sequence (Fig. 2), consistent with their proposed mutational profiles^4^,^28^. The choice of the BER enzyme used in this step provides lesion selectivity; all further steps are the same for each type of lesion. The BER enzymes chosen to study included uracil-DNA glycosylase (UDG) for processing dU (Fig. 3b)^35^; either bacterial formamidopyrimidine-DNA glycosylase (Fpg) or human OG glycosylase 1 (hOGG1) for excising dOG (Fig. 4b; Supplementary Fig. 1)^36^,^37^; human NEIL1 (hNEIL1) for cleaving at dSp (Supplementary Fig. 2)^34^; whereas the AP site lesion can directly proceed to step II of this method for labelling. Reaction times for each BER enzyme processing its substrate were optimized to achieve >99% conversion to product as determined by polyacrylamide gel electrophoresis (PAGE; Figs 3c and 4c). The reaction times were also optimized for each lesion BER enzyme combination (Figs 3b and 4b; Supplementary Figs 1 and 2). The critical difference for each BER enzyme is whether it is a monofunctional (Fig. 3a) or a bifunctional (Fig. 4a) glycosylase, thus determining the precise enzyme(s) used in the next step (Figs 3a and 4a).

### Formation of a gap site at the lesion for marker insertion

Monofunctional glycosylases, such as UDG, yield an AP site in an intact DNA strand as the product^38^,^39^, requiring further processing by APE1 to furnish the desired single-nucleotide gap with a 3′-OH on the 5′-oligomer and a 5′-phosphate on the 3′-oligomer, ready for a polymerase to insert the marker nucleotide in the next step (step II; Fig. 3a)^39^. The intact AP sites were cleaved by adding APE1 directly to the reaction from the previous reaction. By following APE1 activity by PAGE, we found a 1 h incubation to yield the desired product in nearly quantitative yield (Fig. 3b). For bifunctional BER enzymes, such as Fpg^40^, hOGG1 (ref. 39) or hNEIL1 (ref. 40), the gap site was processed with Endo IV and a 3′-phosphatase to yield the desired flanking functional groups for insertion of the marker nucleotide (Fig. 4a). This enzyme combination is capable of reactions on gap sites that may or may not have a sugar fragment on the 3′-OH on the 5′-side of the gap^39^. Such a fragment may exist if the bifunctional BER enzyme only possesses β-elimination lyase activity that is characteristic of hOGG1 removal of dOG^39^, while Fpg and hNEIL1 both have β- and δ-elimination lyase activity yielding a gap site without the sugar fragment^39^,^40^. In these reactions, Endo IV and a 3′-phosphatase were added directly to the reaction from step I and allowed to react for 2 h. Because bifunctional glycosylases cleave the strand in the previous step, the yield of the gap processing reactions was not directly determined by PAGE; instead, the yield of marker nucleotide incorporation in the next step (step III; Fig. 4a) provides the reaction yield. On the basis of the yield from step III, these two enzymes furnish the desired single-nucleotide gap product in nearly quantitative yield (Fig. 4c).

### Polymerase insertion of the marker nucleotides

Once a gap was generated at the lesion site, a polymerase that was only given dNaMTP or d5SICSTP allowed insertion of the marker in place of the lesion (step III; Figs 3a and 4a). Because only dNaMTP or d5SICSTP exist in the reaction without any other canonical nucleotides, they will be the only nucleotides inserted. Literature precedence has demonstrated that Klenow fragment of DNA polymerase I deficient in exonuclease activity (Kf exo^−^) has the potential to react at a gap site in a duplex, as well as being capable of inserting non-natural nucleotides^41^,^42^; hence, Kf exo^−^ was selected for study of inserting either dNaMTP or d5SICSTP opposite a template dG. On the basis of PAGE analysis (Figs 3b and 4b), Kf exo^−^ with either of the marker triphosphates added to the reaction from step II could insert opposite a template dG to furnish the nicked duplex in a 1-h incubation with a nearly quantitative yield (Figs 3c and 4c). The efficiency of incorporating dNaMTP or d5SICSTP opposite dA, dC or dT were also evaluated and found to be >93% (Supplementary Table 1). This step was the first forcing reaction in the protocol, and the high yields observed were encouraging for the ultimate success of the labelling reaction.

### Sealing the marker at the lesion site via ligation

The duplex product from step III has a nick with a non-natural base pair on the 3′ terminus of the 5′ oligomer (Figs 3a and 4a). Previous studies from our laboratory demonstrated that this side of the nick is the most challenging to seal when a non-native base pair is present^43^; therefore, initial test reactions were set up to ligate either dNaM or d5SICS base paired with a dG on the 5′-side of the nick (step IV). This system allowed optimization of the ligation reaction to yield the desired duplex product. The best conditions found to seal the nick required the addition of dimethylsulphoxide (DMSO, 20% (v/v))^44^, decreased ATP (0.1 mM) and a large excess of T4-DNA ligase (800 units (U)) that were allowed to react for 16 h at 25 °C. These conditions gave a yield up to ∼70%, and further refinement did not achieve higher yields. To complete these studies, tests were conducted with all combinations of dNaM or d5SICS paired with the canonical nucleotides to elucidate if there are any pairing combinations that might be better or worse for ligation (Fig. 2). These studies determined that dNaM or d5SICS paired opposite a dG at the nick site led to the highest ligation yields (∼70%; Supplementary Table 2), and all other cases gave lower yields (∼56–65%; Supplementary Table 2). Finally, a test of the ability to label multiple lesions with this approach was conducted with two dU lesions placed nine base pairs apart. PAGE analysis found the overall ligation yield to be ∼45% (Supplementary Fig. 9). Because high yields (>90%) could not be achieved for the ligation step, an alternative approach was pursued to install the marker nucleotide (dNaM or d5SICS) at the gap resulting from BER activity on multiple lesions.

### Polymerase extension to seal the marker nucleotide

**Figure 5:**
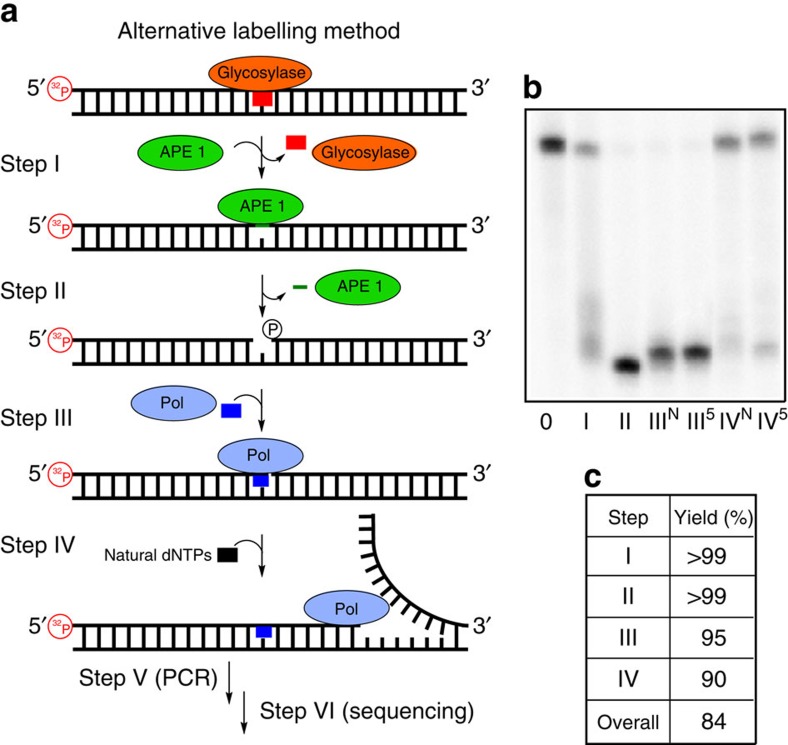
Labelling of a lesion with a marker nucleotide utilizing polymerase extension. (**a**) The alternative scheme for labelling, amplifying and sequencing a lesion. (**b**) PAGE analysis to monitor and quantify enzyme-catalysed labelling reactions on the *KRAS*-U duplex with a U·G base pair (Fig. 2). Reactions were monitored by 5′ ^32^P-labelling of the lesion-containing strand, and the gels were quantified by phosphorimager autoradiography. Step 0 represents the starting strand before reaction. Steps III and IV were conducted with either dNaM or d5SICS, in which the differences are marked with either a superscript N or 5, respectively. (**c**) The percentage of yield for each enzymatic step of the labelling scheme with a template dG.

Ligation at the site of the marker nucleotide was the most challenging step in our scheme with the lowest overall yield (56–72%; Supplementary Fig. 3). Because of the low abundance of lesions in the genome, it is preferred to maximize the yield in all steps. To bypass the ligation reaction, the nicked duplex bearing a marker nucleotide at the lesion site can be subjected to polymerase extension with a strand displacing polymerase, such as Kf exo^−^ (Fig. 5a). The extension reaction will initiate from the marker nucleotide on the 3′-end of the priming strand (Fig. 5a). To conduct this extension, we simply added the four natural dNTPs after completion of step III and allowed Kf exo^−^ to perform the extension reaction. One limitation to this approach is that any product from step III that does not have the marker nucleotide present will also lead to full length duplexes, and a method for bypassing this issue is proposed below. The other limitation to this approach is that only one marker nucleotide per strand, and therefore one lesion per strand, can be detected because during the polymerase extension step any other marker present will be displaced by the polymerase. In spite of this limitation, polymerase extension using the strand with the marker nucleotide on the 3′-end was conducted. On the basis of PAGE analysis (Fig. 5b), the intact duplex was observed in high yield (90%), and the only impurity in this reaction was the presence of strands that failed to extend (10%; Fig. 5c).

### PCR amplification of duplex DNA with a marker nucleotide

The sealed duplex with a dNaM or d5SICS marker nucleotide at the lesion site from step IV was PCR amplified. At this stage, the duplex product can directly be subjected to PCR amplification (Figs 3a and 4a); however, the strand that does not contain the marker nucleotide will be more favourably amplified during this step than the strand with the dNaM or d5SICS marker. For initial analysis of marker nucleotide incorporation, the two strands of the duplex were of different lengths allowing PAGE purification of the marker-containing strand away from the template strand (Fig. 2). Next, the marker-containing strand was subjected to 20 cycles of PCR following literature protocols^22^ to yield a population of amplicons (step V). Locating the position of the UBP within the amplified DNA was determined in the sequencing step (step VI; Figs 3a and 4a).

**Figure 6:**
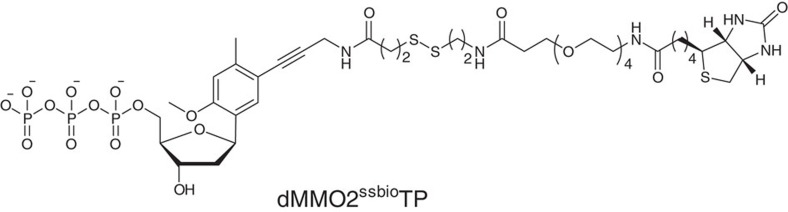
The dMMO2^ssbio^TP unnatural nucleotide utilized for affinity purification. .

For biological samples, the strands will not have different lengths, and PAGE separation of the strands will not be possible. When applying this method, we envision three approaches for purification of the marker-containing strand. (1) Immunoprecipitation of DNA strands containing the lesion of interest, followed by labelling and sequencing. This method is limited by the availability of immunoprecipitation grade antibodies for the lesion of interest. (2) Placement of an affinity purification tag on one of the marker nucleotides to allow purification such as the biotinylated dMMO2^ssbio^TP that can be substituted for dNaMTP as the UBP in this approach^25^. Insertion of d5SICSTP in step III followed by amplification using d5SICSTP and biotinylated dMMO2^ssbio^TP (Fig. 6) provides the affinity purification tag^25^,^45^. As demonstrated below, utilization of the d5SICS·dMMO2^ssbio^ UBP was an excellent approach for purification of the labelled strands. (3) Utilization of α-phosphorothionucleotide triphosphates of dNaM or d5SICS, dNaM^αS^TP or d5SICS^αS^TP, respectively, during step III (Figs 3a and 4a) would incorporate a functional group into duplex DNA that has orthogonal reactivity for purification purposes. Literature precedence has demonstrated reacting phosphorothioate-bearing DNA with iodoacetamide functionalized biotin for labelling to conduct affinity purification^25^. Hence, the use of α-phosphorothionucleotide triphosphates of dNaM or d5SICS during step III would provide a third approach for applying our method to *in vivo* samples, as has been proposed by the Romesberg laboratory^24^. Therefore, we tested this proposal by synthesizing the α-phosphorothionucleotide triphosphates of dNaM (dNaM^αS^TP) following literature protocols^24^ and incorporated it into step III when Kf exo^−^ inserted the marker at the gap generated by the lesion. PAGE analysis determined that dNaM^αS^TP could be substituted for dNaMTP during this protocol (Supplementary Fig. 4).

### Sequencing of the marker base pair in place of a DNA lesion

Once amplicons have been generated with the dNaM·d5SICS UBP located at the lesion site in the original duplex (Figs 3a and 4a), sequencing provides the readout of the base pair's location. In the present iteration, the method for sequencing the UBP was conveniently achieved by the observation of an abrupt stop during Sanger sequencing^45^. Application of this method with either the dNaM or d5SICS nucleotide in the template strand correctly identified the location of the modification (Fig. 3a; Supplementary Fig. 5). As a test of the robustness of the method, the template containing either the dNaM or d5SICS nucleotide was subjected to 20 cycles of PCR before Sanger sequencing. The sequencing chromatograms demonstrated the ability to retain the marker nucleotides in the template strand; however, experiments did show a small level of impurity that was amplified during the PCR workup (Supplementary Fig. 5). These results verify the ability to mark and amplify at lesion sites in a homogenous solution of lesion-bearing oligomers, leading us to question how our method will perform with samples that model those found from biological sources.

### Lesion sequencing in simulated biological contexts

**Figure 7:**
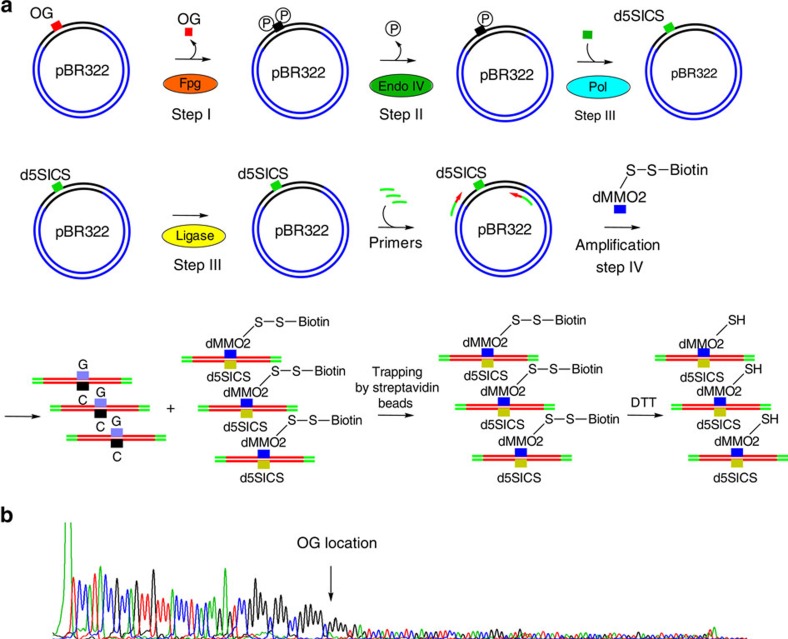
Detection of a dOG lesion in plasmid DNA. (**a**) The scheme comprising dOG labelling by unnatural base followed by amplification. (**b**) Sanger sequencing showing stop of the sequencing indicating the position of the dOG lesion.

Demonstration of this sequencing approach was then performed in a plasmid bearing an dOG nucleotide. Site-specific incorporation of an dOG nucleotide in a plasmid was achieved via an approach developed in the Wang laboratory^46^. The dOG was placed in a the *VEGF* promoter sequence context where a run of dG nucleotides is expected to be a hotspot for G oxidation in cellular DNA^47^. Sequencing of dOG in this context also allowed us to demonstrate the feasibility of using dMMO2^ssbio^TP for affinity purification of the labelled strands. The dOG-containing plasmid was subjected to a one-pot replacement of the lesion by the UBP via the method outlined above. Before Sanger sequencing, the amplicons bearing the UBP were isolated with streptavidin beads from other non-labelled DNA and released by addition of dithiothreitol (DTT) (Fig. 7a). The sequencing chromatogram correctly identified the location of dOG (Fig. 7b). This approach verifies the ability to sequence a lesion within a much larger DNA context.

### Lesion sequencing with a large excess of background DNA

DNA samples from a biological source would have lesions in very low amounts. Next, a test of the feasibility of our approach to find and sequence a lesion with a large background of undamaged DNA was then pursued. Mixtures that contained a dU lesion strand with non-damaged strands in a ratio of 1:10, 1:100 and 1:1,000, respectively, were processed and sequenced to verify that a large background of DNA does not interfere with the processing steps. Again, the dMMO2^ssbio^TP was used to affinity purify the labelled amplicons. The sequencing results illustrated that the method outlined was capable of correctly identifying the location of a lesion in a background of native DNA strands (Supplementary Fig. 8).

### Detection of marker nucleotides by the α-HL nanopore

Although Sanger sequencing is a widely available method, the abrupt stop points by the polymerase encountering the third base pair will not be favourable for identification of more than one marker UBP that might exist in real samples. Therefore, we sought another approach for detecting the marker nucleotides that would have the potential to read more than one marker per strand. Our laboratory has an interest in studying DNA modifications by electrophoretically passing DNA strands through the α-HL protein nanopore embedded in a lipid bilayer^26^,^48^,^49^,^50^,^51^,^52^. The α-HL nanopore analyses one DNA molecule at a time to give ion current versus time traces. Differences in the patterns of these traces allow the analysis of variations between populations of molecules. This feature will be advantageous when determining if more than one modification site exists in a strand of DNA, which cannot be readily achieved by current Sanger sequencing technology. Synthesis of a fifth and sixth dye pair and their incorporation into Sanger sequencing would advance this method; in contrast, the α-HL nanopore is a label-free technique actively being explored for DNA sequencing^27^,^53^.

Free translocation of DNA through the nanopore under an electrical bias is too fast to read the individual nucleotide sequence without the use of molecular motors^9^,^54^. To overcome this limitation, our laboratory has focused on the use of current-modulating adducts for detection of lesions in DNA^26^,^48^,^49^,^55^. Previous studies identified 18-crown-6 to be an excellent current-modulating marker in NaCl electrolyte solutions that allowed detection of one to three of these adducts in a single strand of DNA^26^,^56^, and we therefore adopted our previous method to the detection of the one and two marker nucleotide installed by the previous steps described herein.

**Figure 8:**
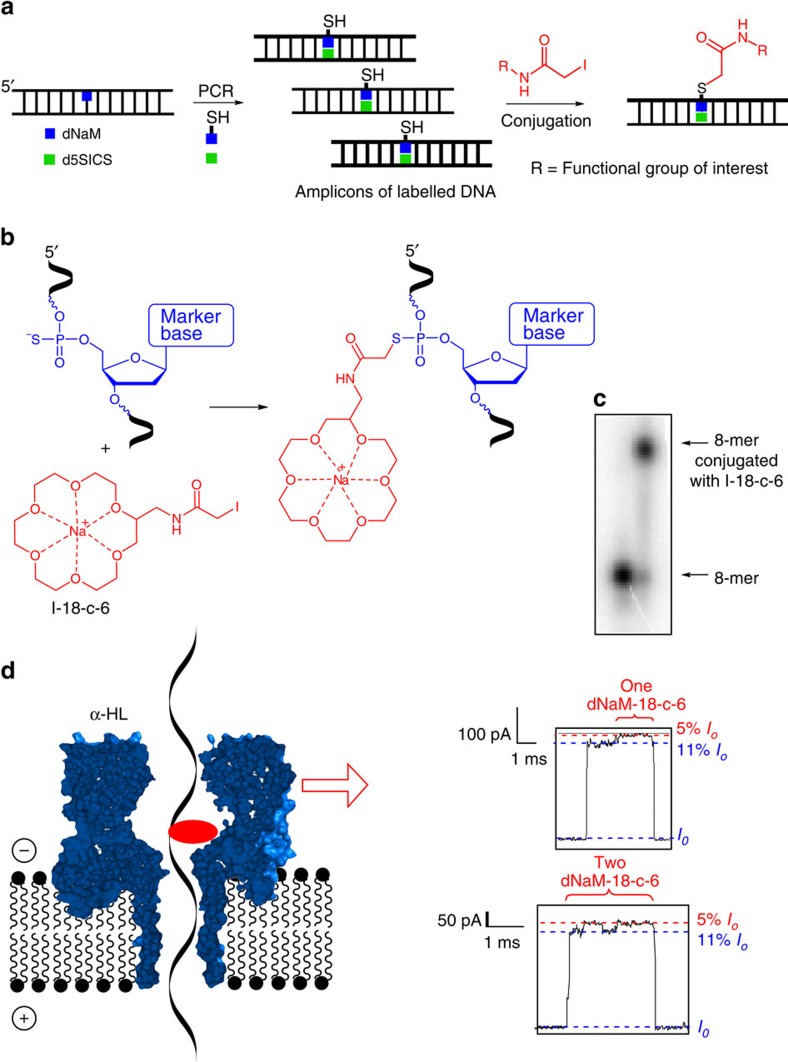
New method for detection of marker nucleotides using the α-HL nanopore. (**a**) PCR amplification with dNaM^αS^TP and labelling of DNA containing phosphorothioate by an iodoacetamide. (**b**) Reaction for phosphorothioate-containing DNA labelling by I-18-c-6. (**c**) Gel-shift analysis of labelled 8-mer by I-18-c-6 to confirm the reaction yield. (**d**) Translocation of DNA (*KRAS*-S^−^; Fig. 2) labelled by I-18-c-6 through the α-HL nanopore providing a modulation in the deep-blockage current level observed as the 11% *I*_*o*_ signal decreasing to 5% *I*_*o*_ that signals the presence of the crown ether and the marker nucleotide (that is, lesion). Nanopore measurements for one 18-c-6-labelled site were conducted at 140 mV bias and for two 18-c-6 labels were conducted at 160 mV (*trans* versus *cis*) bias.

Critical to our adaptation for detecting the dNaM or d5SICS marker nucleotides is the ability to use their α-phosphorothionucleotide triphosphates for introduction of a site-specific sulfur atom that does not interfere with any of the other steps (Fig. 8a). As previously stated, the sulfur can be adducted specifically in high yield with an iodoacetamide. Therefore, we used the dNaM^αS^TP previously reported to incorporate a group that can be site specifically reacted on. Alkylation of the phosphorothioate was achieved with the *N*-iodoacetamide of 2-aminomethyl-18-crown-6 (I-18-c-6), which we also synthesized via a method adapted from literature resources (Supplementary Methods)^57^. On alkylation of the phosphorothioate with I-18-c-6, the strand could be analysed by the α-HL nanopore to detect the ion current modulations diagnostic of the crown ether that also identifies the lesion that was in the original strand from step I (Figs 3a and 4a).

To develop this concept, the alkylation reactions had to be optimized. An 8-mer 5′-ATG^S^CATGC-3′ was synthesized with one phosphorothioate (^S^C) in the backbone that allowed optimization to achieve high yield and characterization of the product resulting from alkylation with I-18-c-6 (Fig. 8b,c). Controls with strands that do not have a phosphorothioate failed to give any detectable product. These observations convinced us all signals observed in the α-HL experiment will result from adduct formation at the phosphorothioate.

The α-HL nanopore platform was then used to analyse the original *KRAS* sequence with one and two phosphorothioates installed at the lesion sites (*KRAS*-S^−^ and *KRAS*-2S^−^ (Fig. 2); Supplementary Fig. 6). This strand flanked the *KRAS* sequence with 25-mer tails of poly-dC to enhance entry of this strand into the α-HL nanopore. After alkylation of this strand with I-18-c-6, it was subjected to analysis by the α-HL nanopore using 3 M NaCl as the electrolyte solution. Entry of the strand into the α-HL channel causes the current to decrease from the open channel value (*I*_*o*_) to a deep blockage level (11% *I*_*o*_) when the DNA spans the channel (Fig. 8d). When the 18-c-6 is driven through the β-barrel, the current recorded becomes more deeply blocked (5% *I*_*o*_), diagnostic of the 18-c-6 traversing the narrowest constriction of the pore and signalling the presence of the marker nucleotide (Fig. 8d; Supplementary Fig. 6). From this study, the modulation of the deep-blockage ion current level was similar to the blockages we previously recorded when 18-c-6 methylamine was adducted to an AP site in a DNA strand^26^. In the last study, two 18-c-6 adducted phosphorothioate sites were installed in a strand followed by α-HL nanopore analysis. In this analysis, two current modulations were observed (Fig. 8d; Supplementary Fig. 6), reproducing the previously published results on a closely related adduct^26^. The present data confirmed that the α-HL nanopore can be an alternative platform for detecting the presence of one or two marker nucleotides installed by this method at lesion sites in DNA strands. The emerging power of other nanopore systems to rapidly sequence DNA provide additional avenues to explore direct sequencing of these UBPs^9^,^54^. Finally, detection of more than one dNaM or d5SICS by Sanger sequencing is not currently achievable; thus, it is anticipated that detection of the dNaM through alkylation of its phosphorothioate with I-18-c-6 by α-HL will provide a platform for counting more than one of these marker nucleotides per DNA strand.

## Discussion

Herein, a method was developed to label a DNA lesion with the dNaM·d5SICS UBP that marks the lesion's location in the primary sequence of DNA. To achieve this goal, a four-step reaction sequence was developed followed by PCR amplification and sequencing (Figs 3a and 4a). The overall reaction yield varied between 50 and 65% (Figs 3c and 4c). A low reaction yield was observed when ligation was used to seal a nicked site in the DNA duplex with dNaM or d5SICS. The ligation yield varied with the canonical base paired with dNaM or d5SICS (Supplementary Fig. 3). This approach will label more than one lesion per strand, as long as they are not too close to prevent the reactions in steps I, II, III and IV. As an alternative, polymerase extension past the incorporated dNaM or d5SICS could label a single lesion site in DNA in a nearly 90% overall yield (Fig. 5c). This alternative approach provides a much needed increase in yield that is required when dealing with the low levels of lesions in the genome. The excellent retention and amplification of the dNaM·d5SICS UBP provides the ability to conduct PCR that effectively allows amplification of lesion-containing DNA. This step is critical in obtaining enough amplicons for performing DNA sequencing, particularly Sanger sequencing. While Sanger sequencing is only capable of identifying one dNaM·d5SICS UBP per duplex^22^,^45^, we demonstrate the use of chemical labels in tandem with the single-molecule profiling capability of the α-HL nanopore to provide the ability to observe multiple markers per strand. This straightforward method can be applied to DNA samples leading to lesion labelling in <24 h when ligation of the marker nucleotide is the approach taken; alternatively, if polymerase extension is used for sealing the marker nucleotide into the DNA, the process can be achieved in about 8 h. This method was developed with commercially available enzymes and instruments; while advantageous, it also limits the ability to mark lesion sites in one strand in quantitative yield, as well as to sequence the markers by single-molecule profiling at single-nucleotide resolution. Nevertheless, future engineering of ligases that efficiently accept UBP at the nick site would provide a much needed boost in the yields for labelling of multiple modifications. Furthermore, nanopore sequencing of genomic data has made enormous strides^9^,^54^,^58^, and adaptation of the present labelling and PCR amplification method with these sequencers will likely be the approach applied for routinely sequencing lesions from biological sources.

The genome is under constant threat by the inherent reactivity of DNA bases and by chemical insults to which they are exposed. These chemical changes lead to mutations, in which the original chemical modification is rarely known, but rather implied based on the mutagenic profile. To address this knowledge gap, we developed a method that enables selective replacement of four types of lesions—uracil, 8-oxo-7,8-dihydroguanine, spiroiminodihydantoin and abasic sites by the dNaM·d5SICS UBP in a one-pot, four-step reaction sequence (Figs 3a and 4a). The elegance of this system lies in the ability to PCR amplify the third base pair, which effectively allows amplification of DNA lesions chosen selectively by BER enzymes for replacement with the marker nucleotides. Moreover, this simple method can easily be extended to other types of lesions or modifications to the genome, as long as a BER enzyme exists to conduct step I of the method as outlined (Figs 3a or 4a, [Fig f4]); for example, lesions/modifications with known glycosylases include thymine glycol (NTHL1)^39^, 3-methyladenine (ALK A)^39^, the T in a T·G mismatch (TDG)^39^ and 5-methylcytosine (ROS 1)^59^, all of which would also be exciting avenues for study by this method. Furthermore, this method avoids competition with native nucleotides to maximize labelling yields without interfering side reactions involving insertion of native nucleotides (for example, due to the ‘A rule'). The sequencing protocol for the dNaM·d5SICS UBP is demonstrated by Sanger sequencing, leading to an abrupt stop in the sequence chromatogram for identification of the base pair's location (Fig. 3a). Accordingly, we further developed chemistry around the dNaM·d5SICS UBP that will permit detection of more than one lesion per strand and allow population analysis of lesion location by the α-HL nanopore method (Fig. 8d). The labelling methodology developed can be applied to questions surrounding the underlying chemical reactions that initiate mutations in the genome leading to disease. For example, mutations in codon 12 of the *KRAS* gene can be probed to determine if dOG or dU are responsible for the mutations observed in lung or colon cancer, respectively^4^,^28^. This information will be paramount in preventative medicine for cancers and other deleterious stress-induced diseases resulting from these mutations. The new α-HL nanopore sequencing methodology developed has the potential to become a robust approach for detecting and sequencing non-native nucleotides that is currently only achievable by SMRT sequencing.

## Methods

### Materials

UDG, Fpg, hOGG1, endonuclease IV, T4-polynucleotide kinase, Klenow(exo^−^) DNA polymerase, T4-DNA ligase and OneTaq DNA polymerase were purchased from New England Biolabs. APE 1 (ref. 60) and hNEIL1-DNA glycosylase^61^ were a kind gift from Professor Sheila S. David. dNaM and d5SICS 2′-deoxynucleotide-5′-triphosphates were synthesized by previously published procedures^24^,^62^. Other chemicals were of molecular biology grade.

### DNA preparation and purification procedures

DNA was prepared from commercially available phosphoramidites (Glen Research, VA) by the DNA/Peptide Core Facility at the University of Utah. The DNA was cleaved and deprotected following the manufacturer's protocol, followed by purification using an ion-exchange high-performance liquid chromatography (HPLC) column running a linear gradient of B from 20 to 100% over 30 min while monitoring ultraviolet absorbance at 260 nm (A=20 mM NaP_i_, 1 M NaCl, pH 7 in 10% CH_3_CN/90% ddH_2_O; B=10% CH_3_CN/90% ddH_2_O, flow rate=3 ml min^−1^). Oligonucleotides were dialysed and water was evaporated. The oligonucleotide concentrations were determined by ultraviolet–visible spectroscopy using the primary sequence to determine the extinction coefficients.

### Site-specific replacement of an uracil lesion

Reaction mixture (50 μl) containing *KRAS*-U (10 pmol), reaction buffer (25 mM HEPES, 10 mM MgCl_2_, 5 mM KCl, 1 mM DTT, 1 mM EDTA) and UDG (1 U) was incubated at 37 °C for 30 min. Then AP endonuclease (1 μl, 150 nM) was added to the reaction mixture, incubated at 37 °C for another 1 h and heated to 95 °C for 10 min. Next, dNaMTP or d5SICSTP (3 μl, 500 μM) and Klenow(exo^−^) DNA polymerase (7 U) were added to the reaction mixture and heated at 37 °C for 1 h. The reactions were quenched by heating to 95 °C for 10 min. Finally, 5 μl of DMSO, 1 μl of 3 mM ATP and 2 μl (800 U) of DNA ligase were added to the reaction and kept at 25 °C for 16 h. For the extension assay, a mixture of natural dNTPs was added in step IV, and the template strand without poly T tails and triethylene glycol capping was used.

### Site-specific replacement of uracil lesions, nine bases apart

A reaction mixture (50 μl) containing *KRAS*-U9 (10 pmol), reaction buffer (25 mM HEPES, 10 mM MgCl_2_, 5 mM KCl, 1 mM DTT, 1 mM EDTA) and UDG (1 U) was incubated at 37 °C for 30 min. Then AP endonuclease (1 μl, 150 nM solution) was added to the reaction mixture, incubated at 37 °C for another 1 h and heated to 95 °C for 10 min. Next, dNaMTP or d5SICSTP (3 μl, 500 μM) and Klenow(exo^−^) DNA polymerase (7 U) were added to the reaction mixture and heated at 37 °C for 1 h. The reactions were quenched by heating to 95 °C for 10 min. Finally, 5 μl of DMSO, 1 μl of 3 mM ATP and 2 μl (800 U) of DNA ligase were added to the reaction and kept at 25 °C for 16 h.

### Dilution study of labelling uracil lesion at different ratios

Reaction mixture (50 μl) containing duplex *KRAS* sequence without lesion (20 μg), *KRAS-U* (2 μg, 200 ng and 20 ng) reaction buffer (25 mM HEPES, 10 mM MgCl_2_, 5 mM KCl, 1 mM DTT and 1 mM EDTA) and UDG (1 U) was incubated at 37 °C for 30 min. Then AP endonuclease (1 μl, 150 nM) was added to the reaction mixture, incubated at 37 °C for 1 h and heated to 95 °C for 10 min. Next, dMMO2^ssbio^TP (3 μl, 500 μM) and Klenow(exo^−^) DNA polymerase (7 U) were added to the reaction mixture and heated at 37 °C for 1 h. The reactions were quenched by heating to 95 °C for 10 min. Finally, 5 μl of DMSO, 1 μl of 3 mM ATP and 2 μl (800 U) of DNA ligase were added to the reaction and kept at 25 °C for 16 h. The biotinylated DNA was trapped by streptavidin beads and sequenced.

### Site-specific replacement of 8-oxoguanosine lesion

The reaction mixture (50 μl) containing *KRAS*-OG (10 pmol), reaction buffer (25 mM HEPES, 10 mM MgCl_2_, 5 mM KCl, 1 mM DTT and 1 mM EDTA) and Fpg-DNA glycosylase (40 U) or hOGG1-DNA glycosylase (20 U) were incubated at 37 °C for 2 or 3 h, respectively. Then endonuclease IV (20 U) was combined with T4-polynucleotide kinase (1 U) and added to the reaction mixture followed by incubation at 37 °C for 2 h followed by heating to 95 °C for 10 min. Next, dNaMTP or d5SICSTP (3 μl, 500 μM) and Klenow(exo^−^) DNA polymerase (7 U) were added to the reaction mixture and heated at 37 °C for 1 h. The reactions were stopped by heating to 95 °C for 10 min. DMSO (5 μl), ATP (1 μl, 3 mM) and DNA ligase (2 μl, 800 U) were added to the reaction and kept at 25 °C 16 h.

### Site-specific conversion of spiroiminodihydantoin lesion

Synthesis of *KRAS*-Sp was achieved by literature protocols and purified via the HPLC method outlined above^63^. Reaction mixture (50 μl) containing *KRAS*-Sp (1 pmol), reaction buffer (20 mM Tris, 10 mM MgCl_2_, 5 mM KCl, 1 mM DTT and 1 mM EDTA) and hNEIL1-DNA glycosylase (100 nM) were incubated at 37 °C for 2 h. Then endonuclease IV (20 U) was combined with T4-polynucleotide kinase (1 U) and added to the reaction mixture, incubated at 37 °C for another 2 h and heated to 95 °C for 10 min.

### Reaction analysis by denaturing PAGE

Aliquots (3 μl) of the reactions were monitored to determine the extent of product formation. Analysis was achieved by placing the samples in Ambion gel loading buffer II in a 1:2 ratio that was analysed on a 20% PAGE (acrylamide/bisacrylamide 19:1, 35% urea) under denaturing conditions (27 mA, 3 h) using TRIS-borate-EDTA (TBE) electrolyte buffer.

### ^32^P-labelling

To the oligonucleotide (6 pmol) sample, 10 × polynucleotide kinase buffer (10 μl), γ-^32^P ATP (15 μCi) and T4-polynucleotide kinase (0.5 μl) was added, and deionized water to a total volume of 100 μl. The mixture was heated at 37 °C for 1 h, and inactivated at 95 °C for 10 min.

### Purification and PCR

Single-stranded *KRAS* oligonucleotides containing dNaM or d5SICS were isolated by denaturing PAGE and eluted by the crash and soak method for 36 h at 37 °C and desalted by a QIAquick nucleotide removal kit. Single-stranded dNaM or d5SICS-labelled *KRAS* oligonucleotides were PCR amplified under conditions that include 1 μM primers, 0.5 nM template, 200 μM dNTPs, 50 μM dNaMTP and d5SICSTP, and 2 U OneTaq DNA polymerase in a 20 μl reaction. dNaMTP can be replaced by dMMO2ssbioTP for preparation of DNA that can be isolated via attachment to biotin, and d5SICSTP can be replaced by dSICS^αS^TP for functionalization by the *N*-iodoacetamide of aminomethyl-18-crown-6 for nanopore detection^26^. The PCR procedure was initiated by denaturation at 95 °C for 5 min followed by 20 cycles of PCR. Each cycle consisted of three steps: denaturation at 95 °C for 30 s, annealing at 50 °C for 30 s and extension at 68 °C for 1 min, and the final extension lasted for 5 min. The PCR products were purified by a 3% agarose gel electrophoresis running Tris-acetate-EDTA (TAE) electrolyte buffer.

### Preparation of α-phosphorothio-dNaMTP (dNaM^αS^TP)

The commercially available (Berry & Associates) dNaM nucleoside (12 mg, 0.04 mmol) was dissolved in dry trimethyl phosphate (50 μl), and the mixture was cooled to 0 °C under a nitrogen atmosphere^25^. Next, PSCl_3_ (16.9 mg, 0.1 mmol) and 2,4,6-trimethyl pyridine were added drop wise and the resulting mixture was stirred at 0 °C for 2 h. Tributylamine (59 mg, 0.32 mmol) and a solution of tributylammonium pyrophosphate (72.9 mg, 0.16 mmol) in dry DMF (310 μl) were added. After 1 h, the reaction was quenched by addition of 0.5 M triethylammonium bicarbonate buffer, pH 7.5 (2 ml). The product was isolated by reversed-phase (C18) HPLC (0–35% MeCN in 0.1 M triethylammonium bicarbonate buffer, pH 7.5) to afford dNaM^αS^TP as a mixture of *R* and *S* diastereomers). Yield 23%. HRMS *m/z*: [m+2Et_3_N]^+^ calcd 733.2454 found 733.2461. ^31^P NMR (162 MHz, D_2_O), ref (H_3_PO_4_): −13.07 to −13.23 (m; γ-P), −26.16 to −26.56 (m; β-P), δ −40.74–41.12 (m; α-P=S).

### DNA sequencing

DNA sequencing was conducted by the DNA Core Facility at the University of Utah using BigDye Terminator v3.1 Cycle Sequencing kit following the manufacturer's protocol. The sequencing reaction mixtures contained 0.1 pmol of analysed DNA and 4 pmol sequencing primer.

### Preparation of 2-iodoacetamidomethyl-18-crown-6 reagent

Iodoacetic acid (28 mg, 0.06 mmol) was dissolved in 0.5 ml of acetonitrile/water (4:1 v/v) at 0 °C. 1-Ethyl-3-(3-dimethylaminopropyl) carbodiimide hydrochloride (32 mg, 0.16 mmol) was sequentially added and the resulting mixture was stirred at 0 °C for 10 min. 2-Aminomethyl-18-crown-6 (59 mg, 0.20 mmol) was added and the resulting mixture was stirred at 0 °C for an additional 1 h. Finally, the crude product was extracted to dichloromethane and purified by reversed-phase HPLC running acetonitrile water containing 0.1% (v/v) trifluoroacetic acid (TFA) as the mobile phase to yield the final product. Yield 32%; HRMS *m/z*: [M+Na]^+^: calcd 484.0808 found 484.0811.

### Post-amplification DNA labelling

For backbone labelling, dsDNA with a backbone phosphorothioate (*KRAS*-S^−^: 5′-C_25_ACTCTTGCCTACGCCAXCAGCTCCAACTACCAC_25_-3′ KRAS-2S^−^: 5′-C_25_T**X**CTGAATTAGCTGTATCGTCAAGGCACTCTTGCCTACGCC**A**XCAGC_25_-3′ X=thioate) was incubated with 25 mM 2-iodoacetamidomethyl-18-crown-6 in phosphate labelling buffer for 5 h at 50 °C and the product was purified by ion-exchange HPLC. The reaction with an 8-mer DNA with 18-crown-6 was analysed by 20% PAGE (acrylamide/bisacrylamide 19:1, 35% urea) under denaturing conditions (27 mA, 3 h) using TBE electrolyte buffer. Matrix-assisted laser desorption/ionization–time of flight *m/z*: [M+H]^+^ calculated: 2,781.7; found: 2,781.4.

### Ion channel recording

A custom-built, high-impedance, low-noise amplifier and data acquisition system, designed and constructed by Electronic BioSciences (EBS), San Diego, CA, was used for the current–time (*i*–*t*) recordings. Approximately, 10 μM of DNA was added, and >1,000 events were collected for each voltage with a 100-kHz low-pass filter and at a 500-kHz data acquisition rate. The composition of the buffered electrolyte solution was 3.00 M NaCl, 25 mM Tris and 1 mM EDTA (pH 7.9).

### Ion channel measurements

The glass nanopore membrane (GNM; with radius 800 nm) was fabricated as previously reported^64^. 1,2-Diphytanoyl-*sn*-glycero-3-phosphocholine (DPhPC) bilayers spanning across the orifice of the GNM were prepared^65^. The protein α-HL was diluted to a 1 mg ml^−1^ solution in ultra-pure water (18 MΩ·cm) and the DPhPC was dissolved in decane to a concentration of 10 mg ml^−1^, both of which were stored at −80 °C. A pipette holder with a pressure gauge and a 10-ml gas-tight syringe were used to attach the GNM to the DC system. Two Ag/AgCl electrodes were positioned inside and outside of the GNM to apply a voltage. A plastic pipette tip was used to paint the DPhPC solution (1 μl, 10 mg ml^−1^) on the GNM surface. After addition of monomer α-HL (0.2 μl, 1 mg ml^−1^), a pressure was applied to form a suspended bilayer, followed by reconstitution of a single α-HL nanopore in the bilayer.

### Insertion of *VEGF* promoter sequence to plasmid DNA

The pBR322 plasmid (1 μg) was subjected to restriction-free cloning using 5′-CCGCCAGTTGTTTACCCTCACAAGAGTCCGGGGCGGGCCGGGGGCGGGGTGAGTCCATCACTCGAGCGTTCCAGTAACCGGGCATGTT-3′ and 5′-AACATGCCCGGTTACTGGAACGCTCGAGTGATGGACTCACCCCGCCCCCGGCCCGCCCCGGACTCTTGTGAGGGTAAACAACTGGCGG-3′ primers (1 μM), dNTPs (200 μM), Phusion high-fidelity DNA polymerase (2 U) in a 20-μl reaction. Each cycle consisted of an initial denaturation at 95 °C for 3 min, followed by 20 cycles. Each cycle consisted of three steps: denaturation at 95 °C for 45 s, annealing at 55 °C for 30 s and extension at 72 °C for 1.5 min, terminating with a final extension for 5 min. The product plasmid was analysed by a 1% agarose gel electrophoresis running TAE buffer. The plasmid DNA was purified with an UltraClean PCR clean-up kit from MO BIO laboratories, Inc. The purified plasmid DNA was transformed to competent *Escherichia coli* multiplied and isolated with a Qiagen plasmid maxi kit.

### Insertion of *VEGF* promoter sequence bearing dOG to plasmid

Purified pBR322 plasmid (500 ng) was treated with Nt.Bst.NBI nicking endonuclease (10 U) in a 20-μl reaction for 1 h. Next, the complementary strand 5′-GATGGACTCACCCCGCCCCCGGCCCGC-3′ that hybridizes to the target region was added to the reaction mixture and heated to 80 °C for 3 min followed by cooling on ice for 2 min. This cycle was repeated four times. The resulting gapped plasmid was purified with an Amicon Ultra 0.5-ml centrifugal filter with a 100-kDa cutoff washing by T4-DNA ligase buffer. The oligonucleotide 5′-GCGGGCCGGGGGC**O**GGGTGAGTCCATC-3′ bearing the 8-oxo-7,8-dihydro-2′-deoxyguanosine nucleotide (*O*) was annealed with the gapped plasmid by heating it to 80 °C and slow cooling. Next, T4-DNA ligase (400 U) was added to the reaction and the reaction progressed for 1 h. The ligated pBR322 plasmid containing *O* in the VEGF promoter sequence was purified by agarose gel (1%) that was electrophoresis with TAE buffer for 45 min.

### Detection of an dOG in plasmid DNA

The reaction mixture (10 μl) containing the pBR322 plasmid with an OG lesion (50 ng), reaction buffer (25 mM HEPES, 10 mM MgCl_2_, 5 mM KCl and 1 mM EDTA) and Fpg (5 U), endonuclease IV (10 U) was incubated at 37 °C for 30 min, followed by addition of d5SICSTP (3 μl, 500 μM). Finally, ATP (0.5 μl of 2 mM) and 1.5 μl (600 U) of DNA ligase were added to the reaction and kept at 25 °C for 2 h. The processed pBR322 plasmid (2 μl from the previous reaction) was PCR amplified using forward primer: 5′-CCCTGAGTGATTTTTCTCTGGTCCCGCCGC-3′ (1 μM), reverse primer: 5′-ACGAGAGAGGATGCTCACGATACGGGTTAC-3′ (1 μM), natural dNTPs (100 μM), d5SICSTP (100 μM), dMMO2^SSBIO^TP (100 μM) and OneTaq DNA polymerase (2 U) in a 20-μl reaction. The PCR procedure consisted of initial denaturation at 95 °C for 2 min, followed by 20 cycles of PCR. Each cycle consisted of three steps: denaturation at 95 °C for 45 s, annealing at 55 °C for 30 s and extension at 72 °C for 4 min, and a final extension for 5 min terminated the PCR. The PCR products were purified by 2% agarose gel electrophoresis using TAE buffer. Purified biotinylated DNA was trapped by streptavidin beads (streptavidin magnetic particles, Roche Diagnostics), eluted by DTT (30 mM), and purified with a Qiagen nucleotide removal kit. DNA Sanger sequencing was conducted using BigDye Terminator v3.1 Cycle Sequencing kit.

## Additional information

**How to cite this article**: Riedl, J. *et al*. Identification of DNA lesions using a third base pair for amplification and nanopore sequencing. *Nat. Commun.* 6:8807 doi: 10.1038/ncomms9807 (2015).

## Supplementary Material

Supplementary Information Supplementary Figures 1-9 and Supplementary Tables 1-2
