# Supplementary material for: Identification of DNA lesions using a third base pair for amplification and nanopore sequencing
Source: Nat Commun. 2015 Nov 6;6:8807. doi: 10.1038/ncomms9807 (PMC4667634; doi:10.1038/ncomms9807)
Supplement: Supplementary Information — Supplementary Figures 1-9 and Supplementary Tables 1-2 [file ncomms9807-s1.pdf]

Supplementary Figures

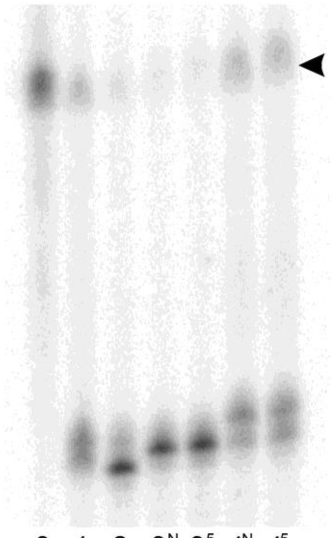

Supplementary Figure 1. A denaturing PAGE gel depicting samples isolated at each step during labeling of an 8-oxoguanosine lesion by the unnatural base using hOGG1 glycosylase. The samples depicted are as follows: lane 1, *KRAS*-OG; lane 2, hOGG1 treatment; lane 3, Endonuclease IV treatment; lane 4, dNaM incorporation; lane 5, d5SICS incorporation; lane 6, T4-DNA ligase treatment (dNaM-labeled DNA); and lane 7, T4-DNA ligase treatment (d5SICS-labeled DNA).

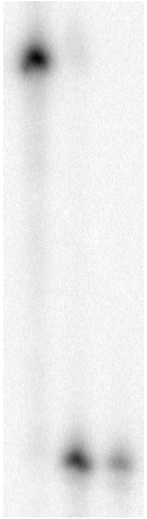

Supplementary Figure 2. A denaturing PAGE depicting samples isolated at each step during conversion of spiroiminodihydantoin lesion to a single nucleotide gap by hNEIL1 DNA glycosylase. The downstream reaction steps are the same as conducted for 8-oxoG and uracil lesions. The samples depicted are as follows: lane 1, *KRAS*-Sp; lane 2, hNEIL1 treatment; lane 3, endo IV + T4-Polynucleotide kinase treatment.

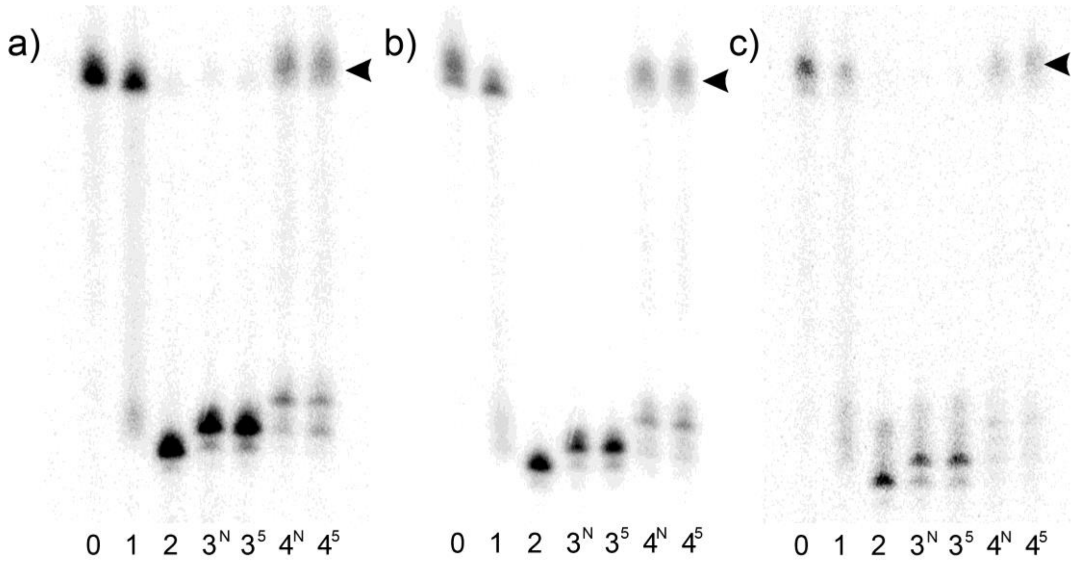

Supplementary Figure 3. PAGE analysis to determine the ability to insert dNaM or d5SICS opposite dA, dC, and dT. a) A denaturing PAGE depicting samples isolated at each step during labeling of a uracil lesion opposite an adenine base by the unnatural base. (b) A denaturing PAGE depicting samples isolated at each step during labeling of a uracil lesion opposite a thymine base by the unnatural base. c) A denaturing PAGE depicting samples isolated at each step during the labeling of a uracil lesion opposite a cytosine base by the unnatural base. The samples depicted in all gels are as follows: lane 1, *KRAS*-U; lane 2, UDG treatment; lane 3, APE 1 treatment; lane 4, dNaM incorporation; lane 5, d5SICS incorporation; lane 6, T4-DNA ligase treatment (dNaM-labeled DNA); and lane 7, T4-DNA ligase treatment (d5SICS-labeled DNA).

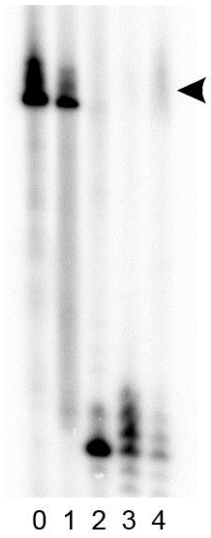

Supplementary Figure 4. Denaturing PAGE depicting samples isolated at each step during labeling of a uracil lesion by dNaM bearing α-phosphorothioate moiety. The samples depicted are as follows: lane 0, *KRAS*-U; lane 1, UDG treatment; lane 2, APE1 treatment; lane 3, dNaM$^{\alpha S}$TP incorporation; lane 4, T4-DNA ligase treatment.

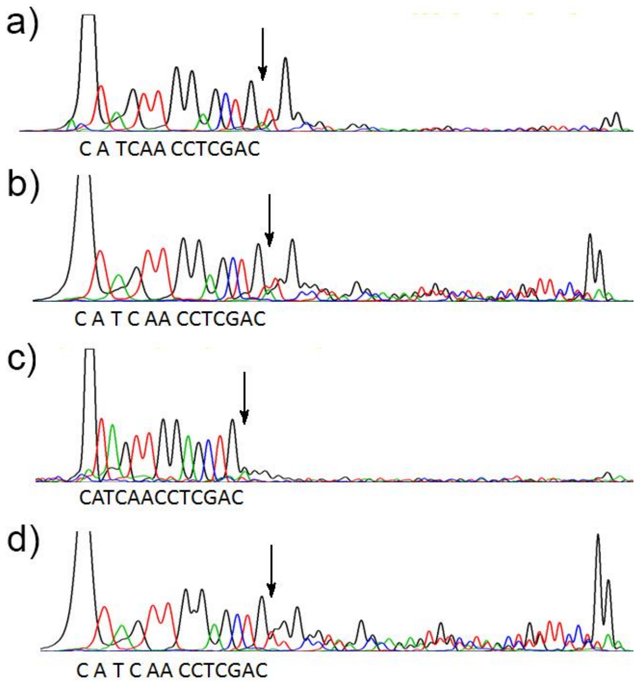

Supplementary Figure 5. Sanger sequencing chromatograms for identification of dNaM or d5SICS in the template strand before and after PCR. (**a**) Sequencing of the *KRAS* sequence with dNaM at the lesion site prior to PCR amplification. (**b**) Sequencing of the *KRAS* sequence with dNaM after 20 cycles of PCR. (**c**) Sequencing of the *KRAS* sequence with d5SICS prior to PCR amplification. (**d**) Sequencing of the *KRAS* sequence with d5SICS at the lesion site after 20 cycles of PCR. Termination of the sequencing chromatogram indicates the position of the marker base, which is indicated by the position of the arrow. The color code for the nucleotides is A = red, T = green, C = black, and G = blue.

a)

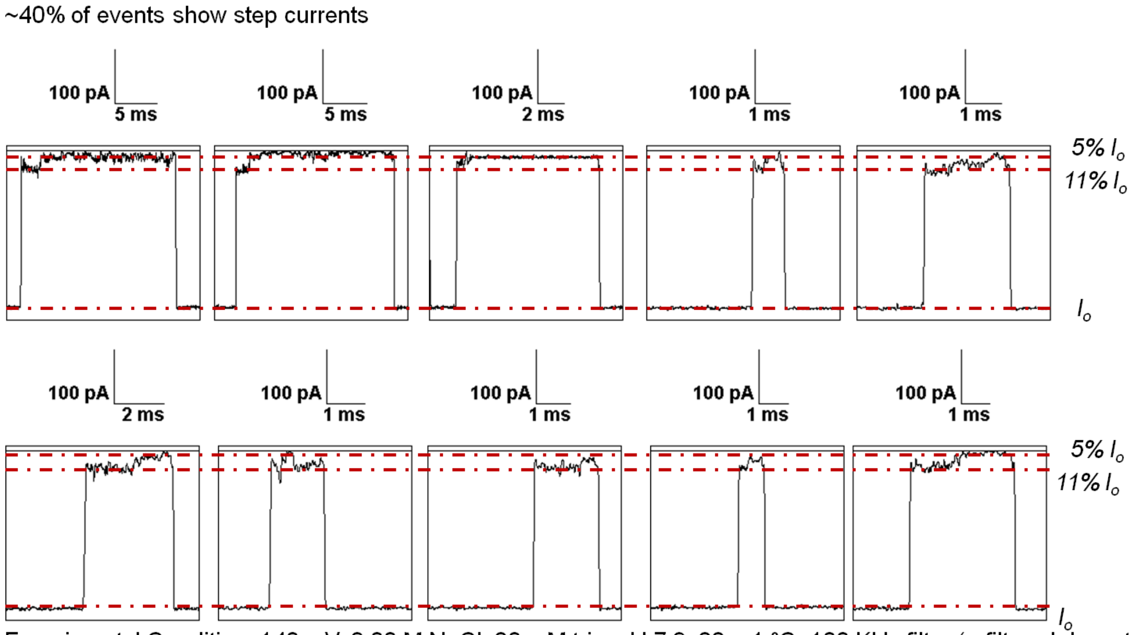

Experimental Condition: 140 mV, 3.00 M NaCl, 20 mM tris, pH 7.9, 22 ± 1 ºC, 100 KHz filter (refiltered down to 10 KHz) I_o = 285 pA

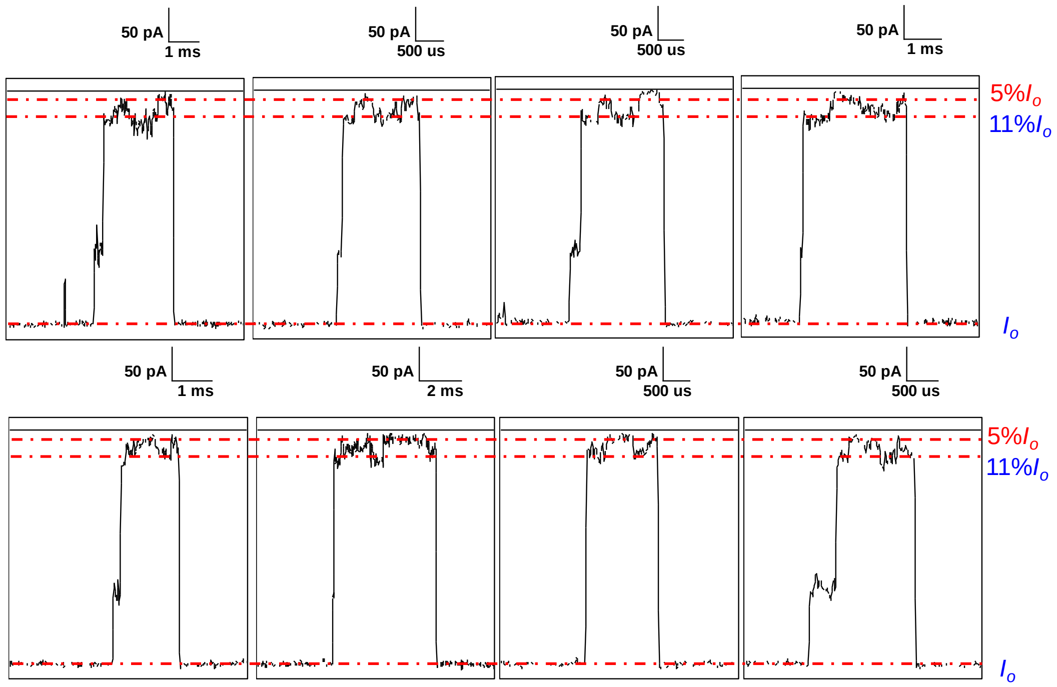

b)

Supplementary Figure 6. a) Representative ion current vs. time traces for analysis of I-18-c-6 adducted DNA strands with the α-HL nanopore. The representative data show modulations of the deep blockage current level from 11% I_0 to 5% I_0 that signals the presence of the crown ether adduct. b) Representative ion current vs. time traces for analysis of DNA strands conjugated with 2 adducts of 18-c-6 with the α-HL nanopore. The representative data show modulations of the deep blockage current level from 11% I_0 to 5% I_0 that signals the presence of crown ether adducts. For the two adduct case, two current modulations were observed in >20% of the traces at 160 mV where 5' vs. 3' entry of the strand is approaching 1:1. With addition of adaptors to bias towards 5' entry only, these observations would be ~50% or greater.

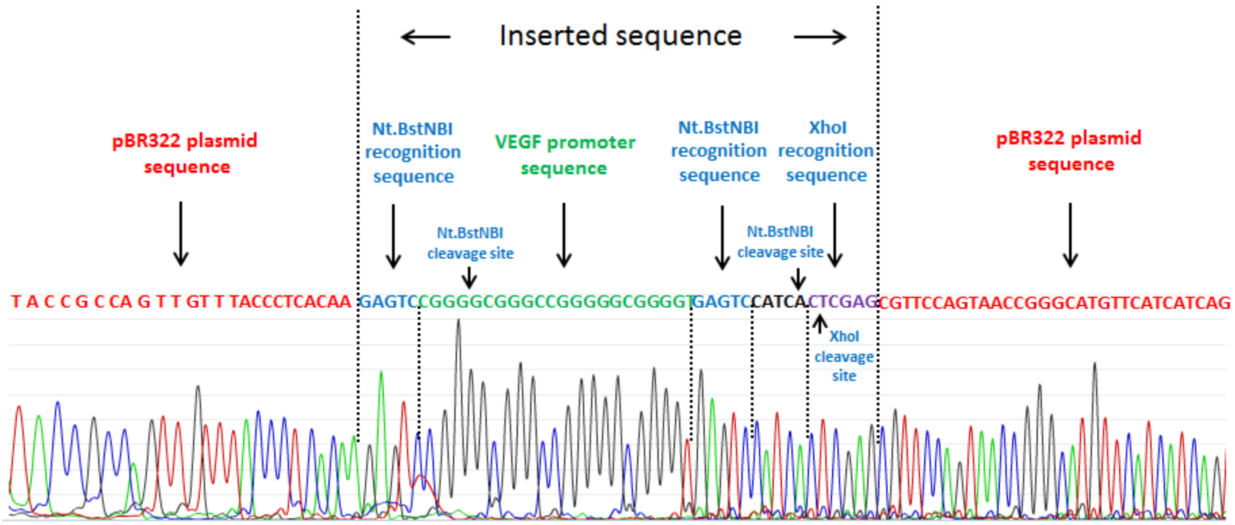

Supplementary Figure 7. Sequencing of the pBR322 plasmid with inserted *VEGF* promoter sequence and the recognition sequence for Nt.BstNBI nicking endonuclease. The color code for the nucleotides is T = red, A = green, G = black, and C = blue.

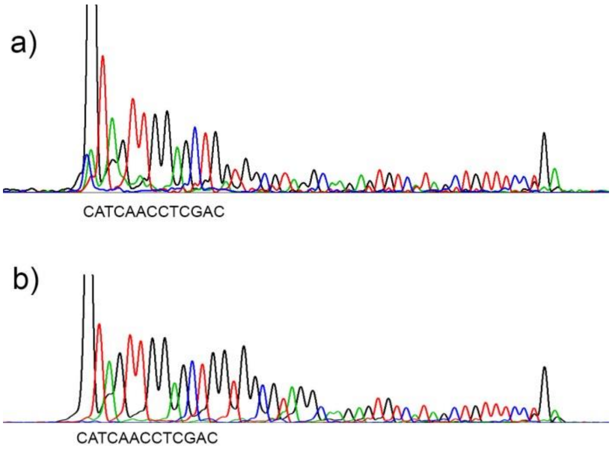

Supplementary Figure 8. Sanger sequencing chromatograms for identification of uracil lesion after labeling by dMMO2^{ssbio}TP in various dilution (**a**) Sequencing of the *KRAS* sequence with dMMO2^{ssbio} at the lesion site at dilution 1:10. (**b**) Sequencing of the *KRAS* sequence with dMMO2^{ssbio} at the lesion site at dilution 1:100. The color code for the nucleotides is A = red, T = green, C = black, and G = blue.

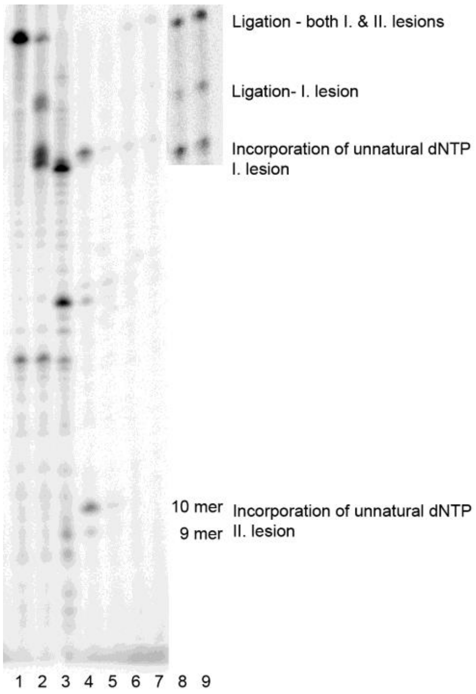

Supplementary Figure 9. PAGE analysis monitoring enzyme-catalyzed labeling reactions of two uracil lesions on the *KRAS*-U duplex with a U•G base pair positioned 9 bp apart representing 1 turn of a DNA helix. The samples depicted in the gel are as follows: lane 1, starting oligonucleotide; lane 2, UDG treatment; lane 3, APE1 treatment; lane 4, dNaMTP incorporation; lane 5, d5SICSTP incorporation; lane 6, T4-DNA ligase treatment (dNaM-labeled DNA); and lane 7, T4-DNA ligase treatment (d5SICS-labeled DNA); lane 8, T4-DNA ligase treatment (dNaM-labeled DNA) – intensified, 40% yield of double ligation; and lane 9, T4-DNA ligase treatment (d5SICS-labeled DNA) – intensified, 45% yield for double ligation.

Supplementary Tables

	Template Nucleotide			
Marker Nucleotide	dG	dC	dT	dA
dNaMTP	96%	95%	94%	93%
d5SICSTP	97%	94%	95%	94%

[a]The average error on each value is ± 5 %. N_{rep} = 3

Supplementary Table 1. The percent incorporation of dNaMTP or d5SICSTP opposite each of the four native nucleotides by Kf exo^{-}.[a]

	Base Pairing Partner			
Marker Nucleotide	dG	dC	dT	dA
dNaM	72%	58%	63%	61%
d5SICS	70%	61%	62%	56%

[a]The error on each experimental value is ± 20%. N_{rep} = 3

Supplementary Table 2. The percent yield for ligation of a nick site with the marker nucleotides dNaM or d5SICS opposite each of the four canonical nucleotides on the 5'-side.[a]
